# Atomic-Scale Imaging of Lithium Vacancies in a Battery Cathode by Multislice Electron Ptychography

**DOI:** 10.1021/acs.nanolett.6c00786

**Published:** 2026-05-29

**Authors:** Dasol Yoon, Harikrishnan KP, Eleanor Richard, Yu-Tsun Shao, Yao Yang, Hector D. Abruña, David A. Muller

**Affiliations:** † Department of Materials Science and Engineering, 5922 Cornell University, Ithaca, New York 14853, United States; ‡ School of Applied and Engineering Physics, 5922 Cornell University, Ithaca, New York 14853, United States; § Mork Family Department of Chemical Engineering and Materials Science, 5116 University of Southern California, Los Angeles, California 90089, United States; ∥ Department of Chemistry and Chemical Biology, 5922 Cornell University, Ithaca, New York 14853, United States; ⊥ Kavli Institute at Cornell for Nanoscale Science, 5922 Cornell University, Ithaca, New York 14853, United States

**Keywords:** multislice electron ptychography, lithium vacancies, battery cathodes, depth sensitivity

## Abstract

Atomic-resolution imaging of battery materials is critical for identification of local defects and structural variations, which are tied to battery performance. However, since battery materials are, by design, optimized to allow ion motion in response to an applied electric field, they are also very sensitive to radiation damage by an electron beam. Image resolution is therefore severely constrained by the dose applied. Here, we show that multislice electron ptychography (MEP) can provide sub-ångström lateral resolution images of both light and heavy elements of a Li-ion battery cathode, along with nanometer-scale depth information and greater dose efficiency than conventional electron microscopy methods. Using the depth-sectioning capability of MEP, we have been able to obtain direct visualizations of Li vacancy clusters, atom column by atom column, in Li_*x*_Ni_0.33_Mn_0.33_Co_0.33_O_2_ (NMC111) cathodes. This capability to track Li distributions will be valuable in understanding, informing, and optimizing electrode material design for ion storage and transfer.

## Graphical abstract

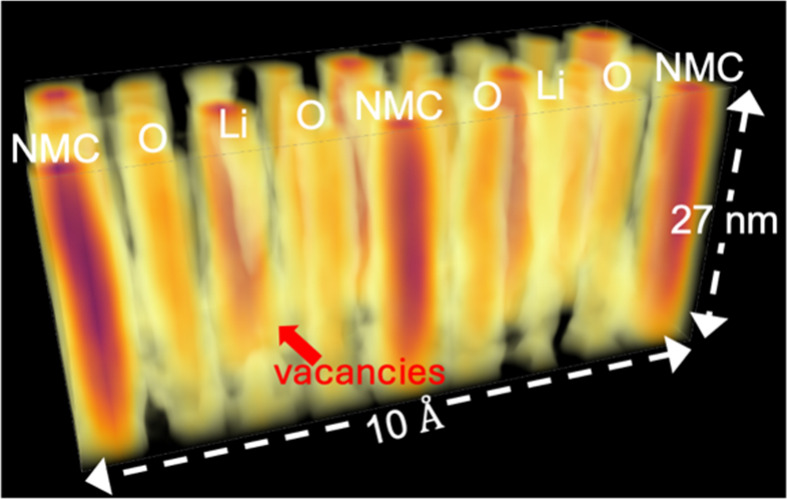

The increasing demand for batteries across sectors such as electronics, electric vehicles, and grid storage highlights the need to develop lower cost and more durable energy storage technologies. Insights into the charge storage and degradation mechanisms could enhance the efficiency, lifespan, and safety of batteries and enable the widespread adoption of sustainable energy solutions.^1,2^ Among them, lithium-ion batteries are widely used for their high energy density, which benefits from the light lithium ions. However, understanding Li (in this manuscript, we use Li to refer to lithium ions, Li^+^) incorporation and diffusion pathways, including variations in Li-site occupancy throughout the charging cycle, remains challenging due to the difficulties associated with simultaneous imaging of both light and heavy elements at the atomic scale^3,4^ and at sufficiently low doses in which radiation damage effects can be recognized and separated from the original elemental distribution.

Nuclear magnetic resonance (NMR) spectroscopy can distinguish local structural information and defects, and its derivative, magnetic resonance imaging (MRI) technique, can spatially resolve the Li distribution in batteries but at a limited resolution of tens of micrometers.^5,6^ Similarly, synchrotron X-ray imaging techniques offer information on the local coordination environment and oxidation states of elements in battery electrodes but spatially averaged over an area typically ranging from tens of nanometers to millimeters.^6,7^ Although these techniques still serve as complementary techniques providing larger length scale information with room for *in operando* experiments in less destructive manners, they lack the localized high spatial resolution information that is required.

Scanning transmission electron microscopy (STEM) enables atomic-resolution imaging that provides local structural information on the sample. Due to the challenge of imaging light atoms near heavy atoms with conventional STEM techniques, imaging modes, such as integrated differential phase contrast (iDPC)^8,9^ imaging, center of mass (CoM)^3^ imaging, and Wigner distribution deconvolution (WDD) ptychography,^4,10^ have been used to visualize Li and transition metals in battery materials at atomic resolution. However, with iDPC and CoM imaging, the apparent position of atoms can be displaced with lens aberrations and multiple scattering in the sample, in addition to other artifacts, such as elongation of atoms and contrast reversals with sample mistilt, thickness, and defocus.^3,11^ Similarly, WDD ptychography also suffers from thickness-induced contrast reversals.^12^

To obtain 3D information, these imaging methods, such as iDPC, need to extract information along the depth direction of the sample by recording multiple images, each at a different probe defocus (one for every depth slice of interest), which is highly dose-inefficient. Additionally, as these imaging modes do not address multiple scattering within the sample, the image contrast is not linear or monotonic with depth, complicating the interpretation of such a through-focal series. With such limited in-depth information, these methods are generally insensitive to structural variations along the beam direction, such as local defects or vacancy clusters. More importantly, the sample must be able to withstand the dose accumulated during the multiple serial acquisitions in the depth-sectioning. As battery materials are beam-sensitive, a more dose-efficient method is desirable.

Multislice electron ptychography (MEP) is a recently developed STEM technique that can reconstruct three-dimensional (3D) structures from a single *x*–*y* scan, with light-atom sensitivity, in a dose-efficient manner.^13^ It addresses the above limitations by utilizing momentum-resolved diffraction patterns recorded at each scan position with a pixelated detector. MEP uses the multislice algorithm to solve the multiple scattering problem^14^ and uses the parallax effect to provide reliable depth information with a typical depth resolution of 2–3 nm.^15^ Some preliminary results on this work were reported as two-page conference abstracts.^16,17^ Subsequently, several studies have already applied MEP to visualize lithium ions and local cation defects in battery cathodes and electrolytes.^18–20^

In this study, we use MEP’s ability to simultaneously image both Li and transition metal atoms in a battery cathode material, to explore the partial occupancy of Li sites and its effects on the neighboring oxygen and transition metal columns. From the 3D structural reconstruction of the sample, we find Li-vacancy clusters buried inside the sample, highlighting the sensitivity of the technique to track the Li distribution at the level of individual Li columns and with a detection sensitivity down to 4 lithium vacancies. This sensitivity limit is set by the maximum dose that can be applied before radiation damage becomes evident, which ultimately sets the achievable resolution limit for imaging electrode materials. Using the 3D imaging capability of MEP, we also identify that degradation layers at the TEM lamella surface have a similar structure as the innate surface reconstruction layers of the cathode particles.

A locally synthesized LiNi_0.33_Mn_0.33_Co_0.33_O_2_ (NMC-111) sample was used as a benchmark material to study variations in local structures and Li distribution.^21^ A cross-section lamella was prepared by focused ion beam (FIB) lift-out from a secondary particle, as shown in the schematic in Figure 1a and Supplementary Figure 1 of the Supporting Information. The lamella contains multiple grains of primary particles, which were previously reported to have surface reconstruction layers (SRLs),^21,22^ labeled here in pink. The surface layer has a rock-salt-like structure, distinct from the trigonal phase of the bulk labeled in blue. Figure 1b is a schematic of the experimental setup for multislice electron ptychography on the prepared lamella. The lamella surface layers are shown in orange to distinguish them from the intrinsic surface of the primary particles. In this work, we reserve the term SRL for the innate surface reconstruction layer and not the FIB lamella surface to avoid confusion.

**Figure 1:**
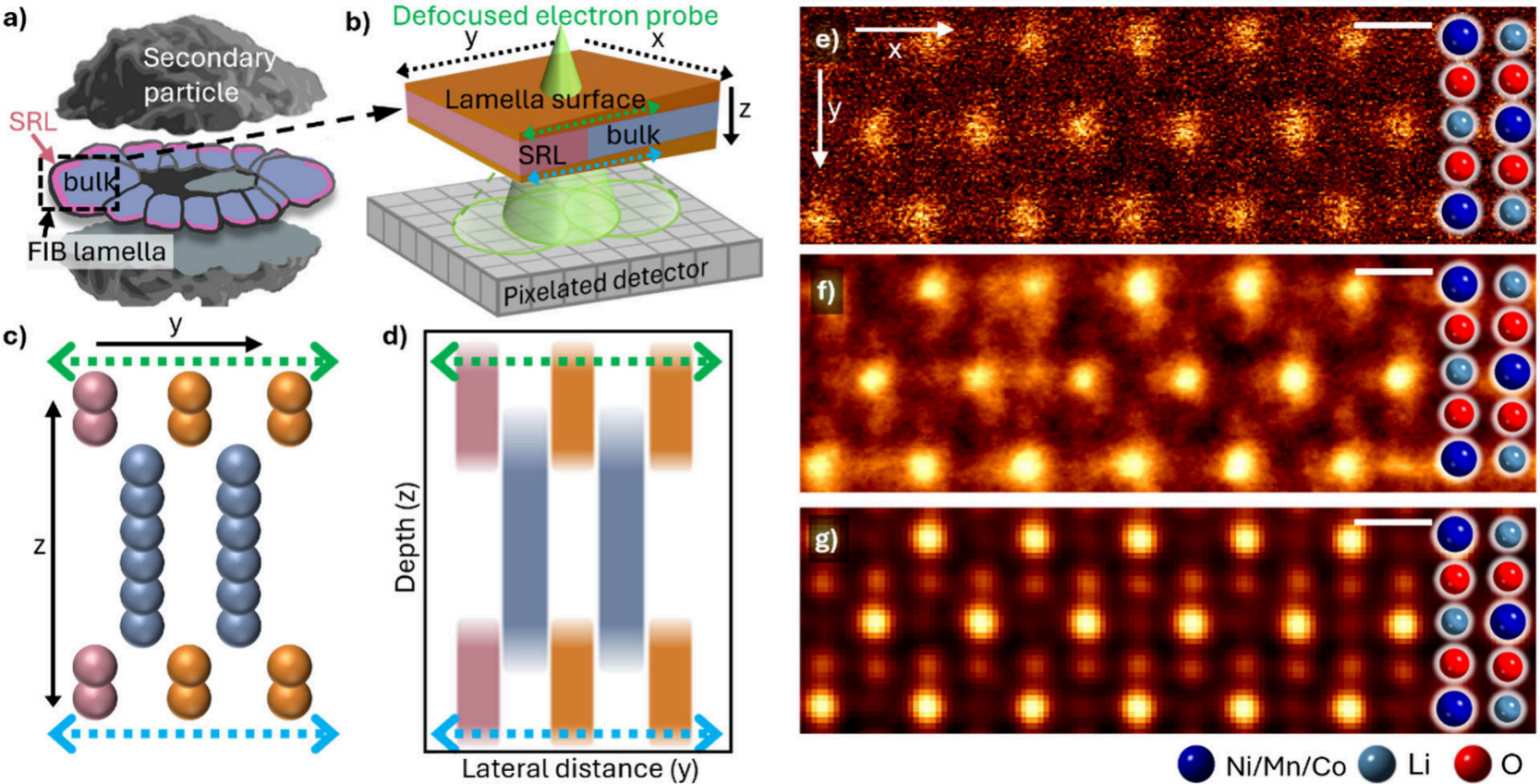
(a) Illustration of a secondary particle of Li-NMC-111, from which the cross-section FIB lamella is prepared. The lamella contains multiple primary particles, whose outer surface reconstruction layers (SRLs, pink) exhibit a phase distinct from the bulk (blue). (b) Schematic of the experimental setup, where a defocused electron beam is used to probe the sample. The diffraction pattern at each scan position is collected on a pixelated detector. The lamella surfaces are shown in orange to distinguish them from the intrinsic SRL of the primary particles. (c) Schematic of atom locations, color coded to indicate whether they are from the SRL, bulk, or lamella surface. The MEP reconstruction of this structure will produce a depth profile like that shown in panel d that captures the structural differences along the *z* direction. Individual atoms are, however, not resolved along the *z* direction due to the limited depth resolution of MEP. The arrows in panels b, c, and d indicate the location of the top (green) and bottom (blue) lamella surfaces. Experimental (e) HAADF, (f) iDPC, and (g) MEP images of Li-NMC-111 along [211], acquired at the same dose, showing the bulk phase of the primary particle. The HAADF image is sensitive only to the heavy transition metal atoms. While the iDPC image is sensitive to the lighter Li and O atoms, the image contrast and spatial resolution are significantly worse than in the MEP image. The MEP image in panel g (sum of the slices along the *z* direction) shows well-resolved Li columns at a spatial resolution unattainable with other techniques. Scale bars are 2 Å.

An overfocused electron probe scans across the specimen to generate a four-dimensional (4D) STEM data set, comprising 2D diffraction patterns collected with a pixelated detector^23^ at each scan position. The 4D data set is analyzed using MEP algorithms^13,24^ to solve the inverse scattering problem and reconstruct the sample structure in three dimensions. The solution takes the form of a series of slices, showing the variations in the sample structure along the depth direction. Stacking the line profiles from these slices results in a depth profile, as illustrated in Figure 1d. Figure 1c shows a schematic of atom locations, and an illustration of a representative depth profile from MEP is shown in Figure 1d. Atoms are resolved laterally (in *xy* dimensions) but not along the depth (*z*) dimension. The depth resolution is limited by the probe depth of focus and dose constraints for beam-sensitive materials. As a guideline, the depth of focus scales approximately as *d*_*z*_ ∼ λ/θ^2^, where λ is the electron wavelength and θ is the probe convergence semi-angle. Increasing θ can therefore improve depth sensitivity but places stricter requirements on residual-aberration correction and alignment. Emerging approaches such as tilt-series ptychographic tomography may further improve depth localization, although the achievable benefit may be constrained by the allowable total dose. Even though the depth resolution (typically 2–3 nm) is not sufficient to resolve individual atoms along the depth (*z*) direction, structural variations on a larger length scale, like differences between the lamella surface and interior, can be resolved. The depth sectioning capability discussed here will be demonstrated later in the paper, through distinguishing the different structures of the pristine interior of the sample from the external layers of the lamella.

The experimental MEP reconstruction slices are summed along the *z* direction to generate a projected image of the sample in Figure 1g. MEP enables us to simultaneously image the light Li and O atoms along with the heavier transition metal elements at higher spatial resolution and contrast when compared to the HAADF and iDPC images acquired at a comparable dose, shown in Figure 1e and f, respectively. The HAADF image can resolve only the heavy transition metal atoms and provides no information about the lighter atoms. While iDPC offers some sensitivity to the light Li and O atoms, the resulting image is still noisy and difficult to interpret due to its higher sensitivity to specimen tilt, thickness, and lens aberrations, including defocus, which is required for obtaining depth information from a through-focal series.

A MEP reconstruction from experimental data of the NMC sample imaged down the [211] zone axis is presented in Figure 2a–d along with simulated MEP reconstructions at a comparable dose in Figure 2e–h. In addition to having only one species in each atom column for easier interpretation, we observe that imaging along the [211] zone axis is more tolerant to knock-on damage than the [100] axis, more commonly used in previous work. This is likely because the [100] direction is along a Li-ion diffusion channel (as visualized in Supplementary Figure 2 of the Supporting Information), while the [211] direction is not. Ptychographic reconstructions along the [100] axis acquired at a similar dose to the [211] axis images display evident effects of knock-on damage that are shown in Supplementary Figure 3 of the Supporting Information. Thus, we recommend imaging down the [211] axis instead of the [100] axis.

**Figure 2:**
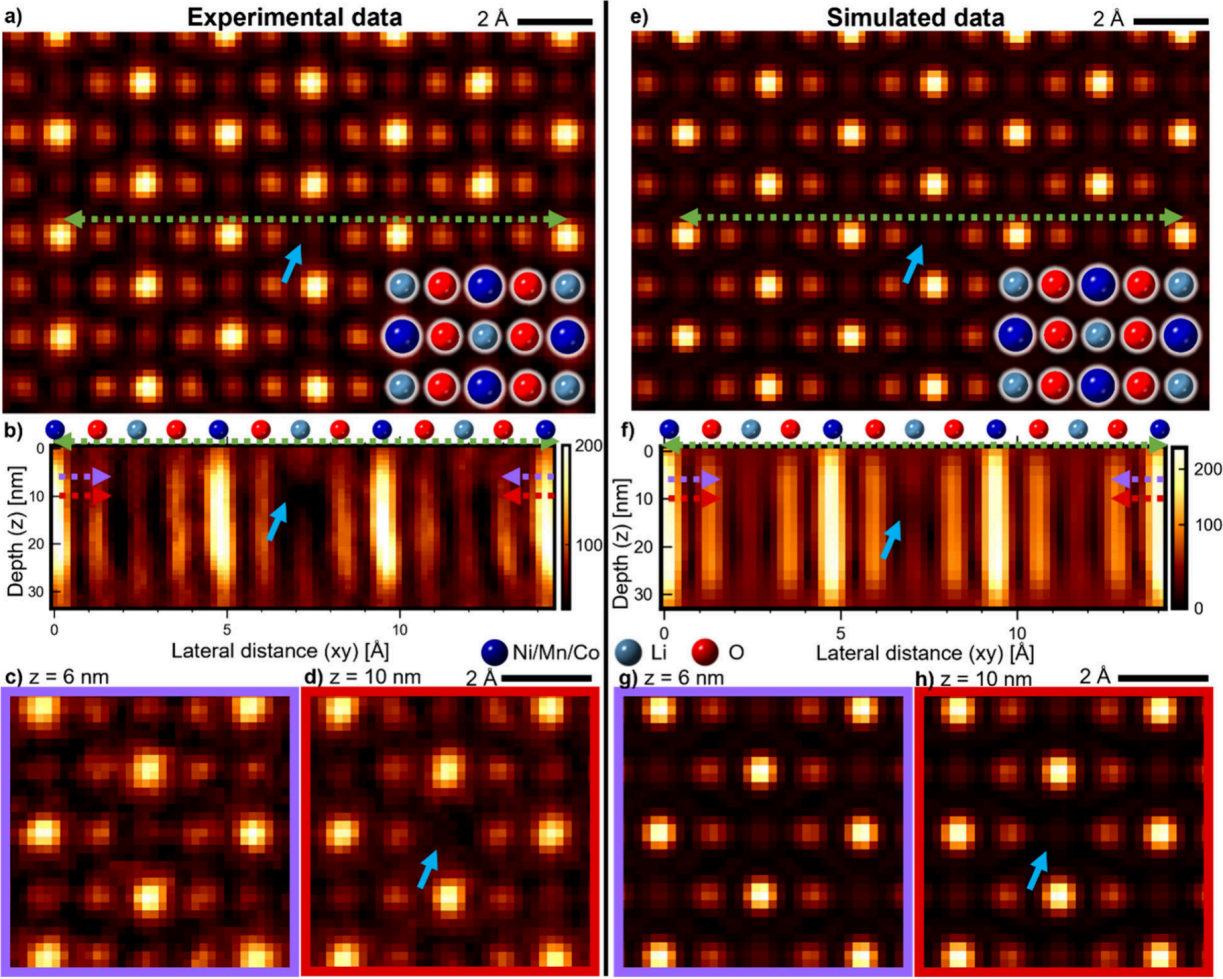
Multislice electron ptychography reconstructions from the experimental data set of Li-NMC-111 (left panel) and simulated data set of LiCoO_2_ (right panel), imaged down the [211] zone axis. (a and e) Sum of the MEP reconstructed layers, where Li columns with vacancies are marked with blue arrows. (b and f) Depth profiles along the row of atomic columns indicated with green dashed lines in panels a and e. Single slices from the MEP reconstruction around the partially occupied Li column, at a depth of (c and g) 6 nm, where the Li site is occupied and marked with a purple arrow in the depth profile, and at (d and h) 10 nm, where there are vacancies on the Li site (blue arrow) and marked with a red arrow in the depth profile. A dose of 8.4 × 10^4^ e^–^·Å^–2^ is used in this data set. The slight tilt visible in the lower half in panel b is due to local specimen bending. No tilt-propagator-based tilt correction was applied to preserve the as-measured 3D morphology.

The projected image obtained by summing up the slices from the MEP reconstruction is shown in Figure 2a and captures both light (Li and O) and heavy (transition metal) atom columns at atomic resolution. As noted in Figure 1, the resolution and contrast in MEP are much higher than for the other imaging techniques. The enhanced contrast and the depth-sectioning capability enable us to quantify the Li distribution within the sample in three dimensions.

Figure 2b shows the depth profile along the green line marked in Figure 2a to visualize variations in occupancies of the different atomic columns along the depth direction. We highlight one Li column (marked with a blue arrow), which shows a significant dip in intensity around 10 nm into the depth direction, indicating the presence of Li vacancies. Characterizing the location and distribution of such Li vacancies is a key step in gaining a deeper understanding of Li diffusion mechanisms and pathways. We further illustrate the presence of this vacancy cluster by showing two slices at different depths in the sample in Figure 2c and d. The slice corresponding to the sample structure at 6 nm shows an occupied Li site, whereas the slice at 10 nm shows a vacant Li site.

To quantify our detection sensitivity to the Li occupancy, we first performed a systematic series of multislice simulations matching the experimental conditions to probe the sensitivity of MEP to detect Li vacancies (Supplementary Figure 4 of the Supporting Information). To simplify the simulations, we approximate the mixed Mn/Co/Ni columns with the averaged atomic element, Co, in the simulations as the scattering factors for the three elements are all extremely similar (Supplementary Figure 10 of the Supporting Information). We find that the unambiguous detection of a vacancy cluster above the Poisson noise limits requires at least four vacant Li sites within the depth resolution of 2–3 nm. That is, a detection sensitivity of 1.3–2 Li vacancies/nm/Li column, with the limit on sensitivity being set by the dose-limited depth resolution. We show the result for four adjacent Li vacancies on the same Li column in Figure 2e–h. These are indicated by the blue arrow in Figure 2e and f. The full series of profiles for 1–4 Li vacancies are shown in Supplementary Figure 4 of the Supporting Information. Individual slices showing the comparison of occupied and vacant Li sites are shown in Figure 2g and h, respectively. Supplementary Figure 11 of the Supporting Information shows that Li vacancies should also be detectable in LiFePO_4_, another commercially relevant Li-ion cathode material. In general, the approach should work for any material where a zone axis can be found that contains atomic columns of only Li atoms in projection. The approach is also applicable to non-Li vacancies; Supplementary Figure 12 of the Supporting Information shows a simulated MEP reconstruction with a prescribed O vacancy configuration, demonstrating detectability under comparable conditions, with sensitivity set by lattice spacing along the beam direction and dose.

Although we use uncycled cathode particles, degradation from long storage under air likely resulted in Li atoms diffusing out of the sample,^25^ creating Li vacancies, in addition to thermal diffusion during annealing.^22^ Based on the ionic conductivity, we estimate the time scale for the Li ions to diffuse to the neighboring lattice point at room temperature (∼900 μs for 3 Å with a diffusion coefficient of 10^–12^ cm^2^/s), which is on the order of the typical time scale for data acquisition (100 μs), while that at a cryogenic temperature is orders of magnitude slower (∼ 10^10^ s for 3 Å; Supplementary Figure 5 of the Supporting Information). With the room-temperature setup, we are likely capturing Li distribution at its equilibrium redistribution within the TEM lamella, but cryogenic temperature preparation and imaging would be more desirable to preserve the Li distribution from the initial material, whether it is cycled or not. We show preliminary reconstructions from data acquired at cryogenic temperatures in Supplementary Figure 6 of the Supporting Information. In Figure 2, we also note that the intensities of the Li columns, relative to the transition metal or oxygen columns, are slightly higher in the experiment than in the simulation. The increased intensity arises from some transition metal atoms occupying the Li sites due to knock-on damage, as the data sets are acquired with a beam energy of 300 kV at room temperature. Although the knock-on damage effects are minimal here, this issue could be further addressed by imaging at a beam energy below the knock-on threshold for the metal atoms (∼80 kV) or at a cryogenic temperature.

Our ability to detect Li vacancies allows us to analyze the relationship between local bond lengths and Li occupancy, as shown in Figure 3. From data sets acquired along the [211] zone axis, such as that in Figure 2, both the projected O–Li–O distance and the neighboring transition metal–oxygen (TM–O) bond length were measured. Analysis of reported crystallographic models of bulk NMC^26^ shows that delitiation is accompanied by a contraction in the O–Li–O distance and an increase in the TM–O bond length along [211], reflecting changes in bond angles and lengths. In Figure 3a, the reconstructed electrostatic potential at Li sites is plotted against the O–Li–O distance (The MEP reconstruction divides the 3D object potential into projected slices. Here, each slice is 1 nm thick; hence, the units are V nm. The potential is dominated by the atomic nuclei; therefore, it is essentially a measure of the number of atoms per nm). A lower object potential, indicating a reduced Li occupancy, is associated with a shorter O–Li–O distance, consistent with the reported bulk behavior. Because beam damage was more pronounced toward the bottom (17–21 nm) of the lamella, as Li atoms on the exit surface were sputtered away by knock-on damage from the electron beam, the fitted trend was derived from the intact upper layers (9–13 nm). Figure 3b plots the O–Li–O distance against the adjacent TM–O bond length, revealing a negative correlation: shorter O–Li–O distances are associated with longer neighboring TM–O bonds. These observations suggest that the ptychography reconstructions capture genuine, localized structural changes rather than reconstruction artifacts, which would be uncorrelated.

**Figure 3:**
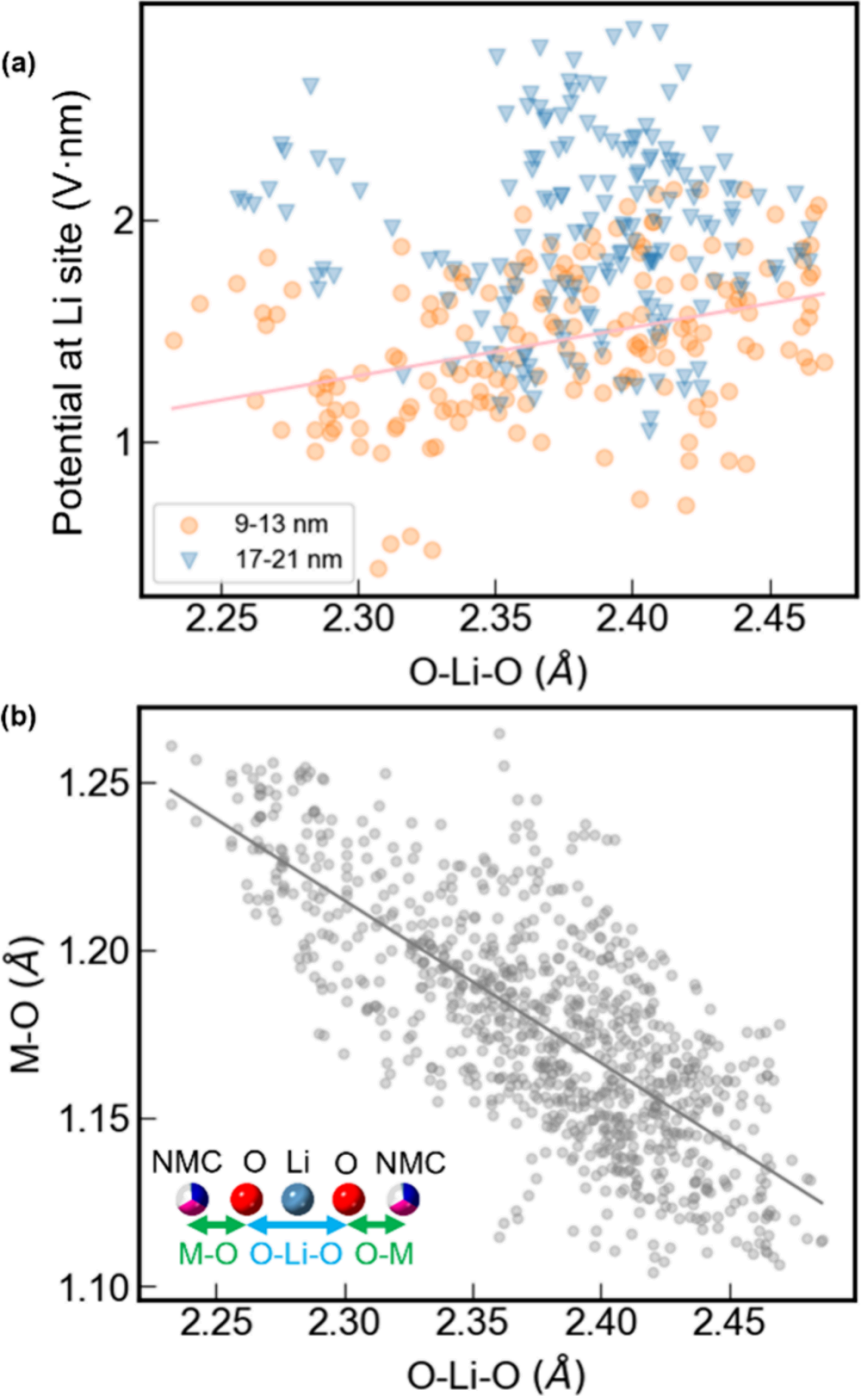
(a) Object potential at Li sites versus projected O–Li–O distance along the [211] zone axis. Lower local Li occupancy is associated with shorter projected O–Li–O distances. To minimize the influence of beam damage, the curve fit was derived from the top four layers (9–13 nm), out of 35 total slices, including vacuum layers. (b) Projected transition metal–oxygen (TM–O) bond lengths plotted against the neighboring projected O–Li–O distance. Regions with shorter O–Li–O distances tend to have longer adjacent TM–O bonds, suggesting local push–pull effects in the lattice.

To assess how accurately MEP can locate Li vacancies in depth, simulations in Figure 4 compare the depth-sectioning capabilities of MEP and iDPC with model structures with and without Li vacancies (Supplementary Figure 4 of the Supporting Information shows the detectability for 1–4 Li vacancies/column). Figure 4a and c shows the MEP and iDPC images of 7.5 nm thick LiCoO_2_ along the [211] zone axis, where the Li column labeled with the white arrow has four continuous Li vacancies at the depth of 2–3.6 nm in the sample. The depth profiles along the row of atoms enclosed in dotted boxes are shown in Figure 4b and d. As MEP solves for the channeling of the electron beam, it provides an accurate depth section of the sample, enabling the identification and quantification of columns with Li vacancies. In comparison, through-focal iDPC imaging struggles to even qualitatively differentiate between the fully and partially occupied Li columns. In addition to the depth profile intensities being noisy and systematically not reflecting the underlying Li distribution, additional multiple-scattering artifacts, such as an offset in the positions of the Li and O columns with respect to their true positions, are also evident in the depth section shown in Figure 4d.

**Figure 4:**
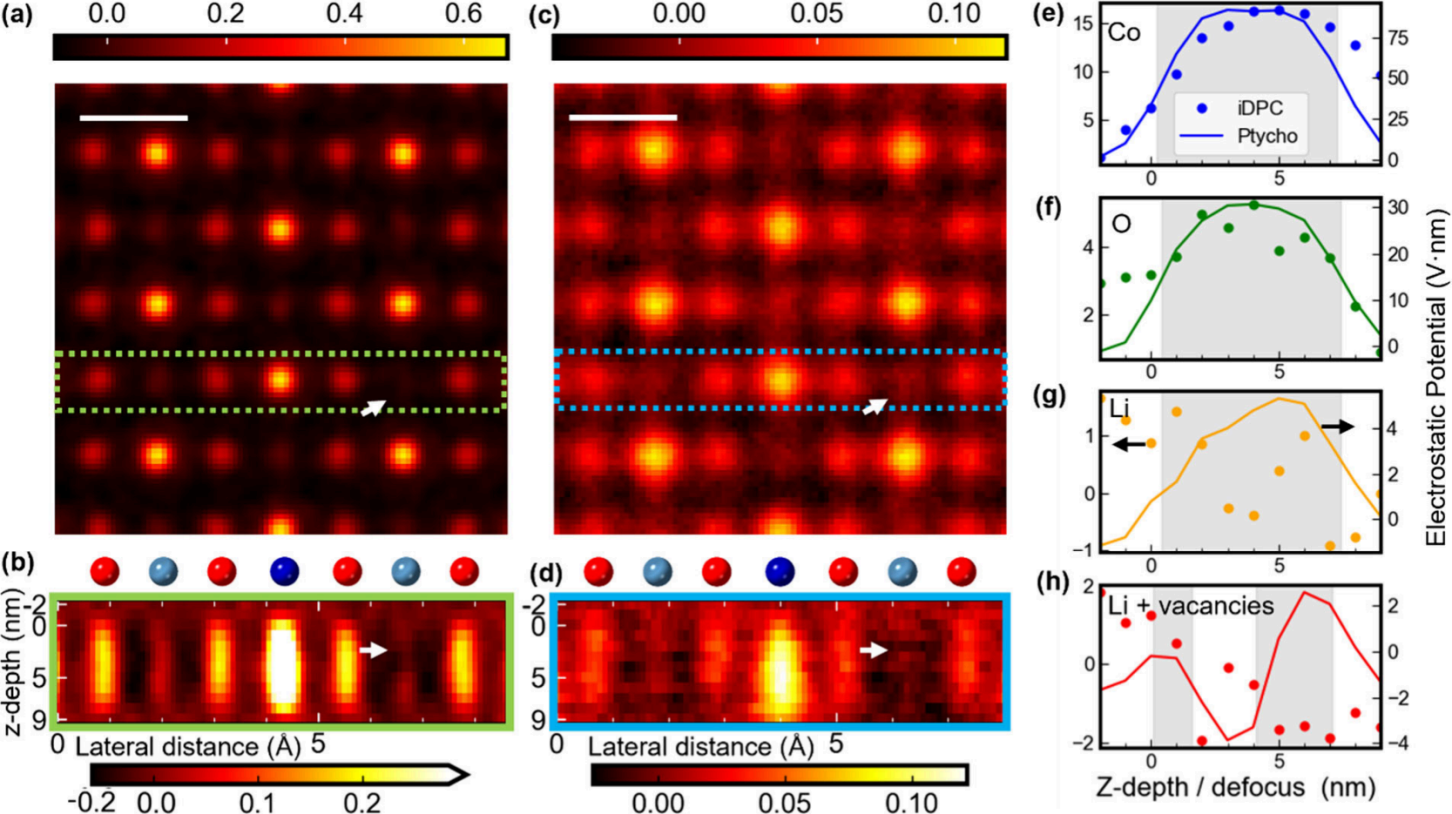
Comparison of simulated (a and b) MEP and (c and d) through-focal iDPC imaging of LiCoO_2_ along the [211] zone axis with the same total dose of 5 × 10^4^ e^–^·Å^–2^. (a) Single image slice at a depth of 3 nm from the top surface in the MEP reconstruction and (c) individual image from the iDPC through-focal series with the probe focused 3 nm below the top sample surface. The location of the Li vacancies are marked with a white arrow, which are more noticeable in MEP than in iDPC. Scale bars are 2 Å. (b and d) Depth sections along the row of atoms enclosed with dotted boxes in panels a and c, where the Li column labeled with the white arrow has four continuous vacancies. The vacancy cluster can be easily distinguished from the occupied sites in the MEP depth profile but not in the iDPC depth profile. The Co column is also incorrectly offset and misplaced with respect to the other atomic columns in the iDPC depth profile. (e–h) Line profiles through the center of individual atomic columns in panels b and d for (e) Co, (f) O, (g) fully occupied Li column, and (h) Li column with vacancies. The gray shaded regions indicate the occupied sites along each column. The iDPC through-focal series provides a low-resolution depth profile for the Co and O columns, while MEP achieves a superior depth resolution as evident from the narrower width of column profiles as shown in panels e and f. For the weakly scattering Li columns, iDPC fails to reliably locate the vacancies in panel h and gives an inaccurate line profile for even the fully occupied column in panel g. In contrast, the MEP profiles are easily interpretable and accurately locate the vacancy cluster.

Figure 4e–h shows the depth profiles of individual atomic columns, Co, O, and fully and partially occupied Li columns, respectively. The gray background indicates the occupied sites. For the Co and O atoms, where both MEP and iDPC give meaningful depth profiles, MEP has a smaller fwhm, indicating a superior depth resolution. MEP provides a smooth profile for the Li columns and a clear measurable distinction between fully and partially occupied columns, while iDPC completely breaks down producing an uninterpretable depth profile.

In addition to the 3D visualization of local atomic defects like the Li vacancies discussed above, MEP also allows us to probe larger scale structural changes, such as differentiating the surface structure from the interior of the sample, as illustrated in Figure 5. This also allows us to separate sample preparation surface damage from the original interior structure. Figure 5a and c shows the rock-salt structure of the sample at the FIB-prepared lamella surfaces (entrance and exit planes of the electron beam indicated with an orange color in the inset). In contrast, Figure 5b shows the anticipated bulk structure with trigonal symmetry (blue color in the inset), evident from the 3-fold rotation axis of the transition metal sites. Moreover, the imaged region is near the surface of the primary nanoparticle, with the left side of Figure 5a–c demarcated by the white dotted line, showing the SRL of the primary particle. This surface structure has been well-studied in the literature,^27^ with a mix of the transition metal and Li atoms equally occupying the cation sites, resulting in a rock-salt structure. We observe that the lamella surfaces (right side of the white dotted line in Figure 5a and c) also possess the rock-salt structure. In FIB sample preparation, we would expect an amorphization layer to form at the surface. The reconstruction of the surface to the rock-salt structure could either be induced by air and moisture exposure of the sample in transfer from the FIB to TEM and/or possibly the electron beam irradiation.

**Figure 5:**
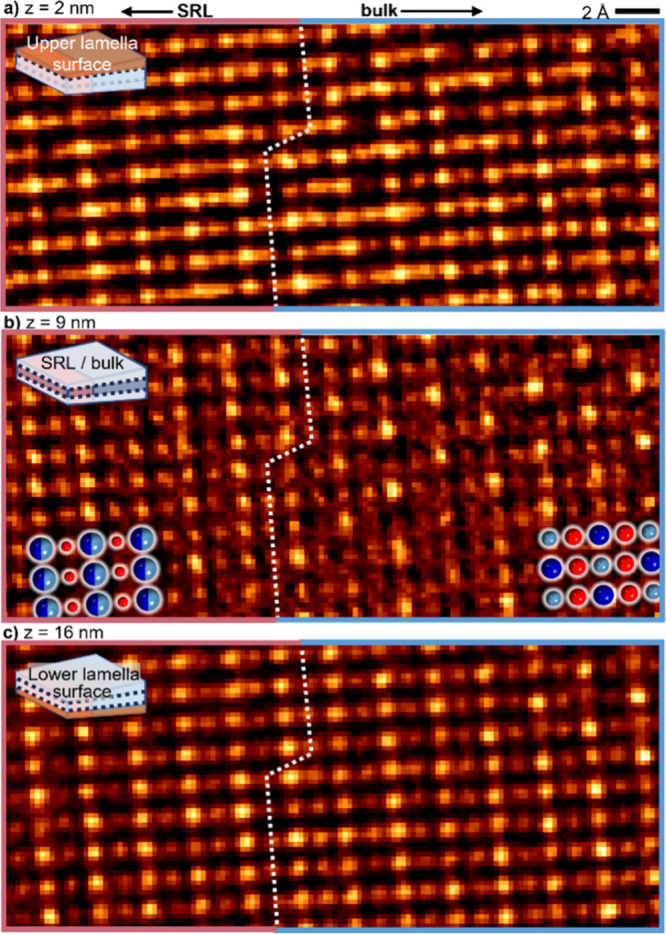
Slices from the experimental MEP reconstruction at three different depths of the NMC sample acquired near the primary particle’s surface or SRL, capturing structural changes along the depth direction. The location from which the slices are obtained are shown in the schematic in the inset. The region to the left of the central white line shows the rock-salt-like innate surface layers, labeled in pink in the schematic and in Figure 1a. The region on the right illustrates where we would expect the bulk region. (a) Top and (c) bottom slices correspond to the lamella surfaces and exhibit the rock-salt-like phase even on the right side, instead of the anticipated trigonal phase of the bulk. Away from the FIB-prepared lamella surfaces, the original SRL/bulk interface is preserved in the (b) middle layer, including the trigonal phase on the right. Some oxygen sites are vacant in panel a.

Away from the top and bottom lamella surfaces, we can still observe the original SRL/bulk interface in the cross-section. Previous studies have reported the propagation of these SRL surface phases to explain the observed voltage fade on cycling.^27,28^ This demonstration of MEP’s ability to identify and map surface and interface structures in three dimensions holds promise for tracking their evolution during cycling studies. While our measurements were not entirely damage-free, the beam damage is systematic, localized, and recognizable. With beam damage minimized under cryogenic temperature, MEP would be crucial in understanding failure mechanisms. Conventional imaging techniques, which are typically most sensitive to the top surface of the sample, would be dominated by the FIB lamellae surface damage rather than the original material, as shown in the case for HAADF imaging in Supplementary Figure 7 of the Supporting Information.

In summary, this work establishes multislice electron ptychography as a proof-of-concept method for probing battery cathode materials with a high sensitivity to atomic-scale vacancies. The detection limit reaches four lithium vacancies, constrained primarily by electron dose. Compared to conventional imaging modes, like iDPC, which often suffer from depth-dependent artifacts and require focal series with inefficient dose distributions, multislice electron ptychography provides artifact-free contrast and dose-efficient acquisition of three-dimensional structural information in a single scan. Depth sectioning further enables a clear separation of surface-modified layers from the intrinsic bulk structure, allowing an analysis of the material in its native state, despite beam sensitivity. These combined advantages emphasize the suitability of ptychography as a dose-efficient and accurate approach for probing ions and characterizing structural heterogeneity in battery cathodes throughout the charging process.

Cross-sectional TEM specimens of NMC-111 were prepared using the standard focused ion beam (FIB) lift-out technique with a FEI Helios G4-UX FIB. The Ga^+^ ion beam energy was sequentially decreased to 2 kV during the final thinning step to reduce damage.

Scanning transmission electron microscopy (STEM) data were taken on aberration-corrected FEI Titan Themis STEM operated at 300 kV with a 21.4 mrad probe forming a convergence angle. The 4D STEM data were acquired using a second-generation electron microscope pixel array detector (EMPAD-G2)^23^ with the following conditions: the beam current of 15 pA, the dwell time per diffraction pattern of 100 μs, 256 × 256 scanning points with a scan step size of 0.364 Å, the camera length of 130 mm, and the collection angle of 55 mrad. These conditions give a dose of 8.4 × 10^4^ e^–^·Å^–2^. A nominal overfocus in the range of 10–20 nm was used.

STEM data shown in Figure 1e–g were taken on a Thermo Fisher Scientific Spectra 300 STEM electron microscope operated at 300 kV with a 30 mrad probe forming a convergence angle. The iDPC data were taken with a Panther STEM detector, and the MEP data were taken with an EMPAD-G2.

Multislice electron ptychography reconstructions were obtained using the fold-slice package^13^ after parameters were chosen via Bayesian optimization and Gaussian processes.^29^ For reconstructions, a slice thickness of 1 nm and 3–4 probe modes were used. The regularization along *z* was initialized at 0.7 for a couple hundred iterations and relaxed to 0.5 for later optimization. Reconstructions were run for up to ∼1000 iterations for experimental data sets and about 400 iterations for simulated data sets. Additional documentation on how to run MEP and its optimization and post-processing is available at https://ptyrad.readthedocs.io/en/latest/index.html. No tilt-propagator-based tilt correction was applied; reconstructions are shown as acquired to preserve local specimen geometry. The raw 4D STEM data set and example reconstruction conditions are available on Zenodo^30^ under DOI: 10.5281/zenodo.19703257.

The simulated 4D STEM data of NMC-111 were generated using the multislice algorithm from abTEM software with parameters matching the experimental conditions.^31^ A Poisson noise equivalent to 5 × 10^4^ e^–^·Å^–2^ was added to each of the simulated data sets. Figure 4 was simulated using a probe forming a convergence angle of 30 mrad.

## Supplementary Material



## Notes

Additional STEM images, simulations, and MEP reconstructions (PDF)
