# Supplementary material for: Atomic-Scale Imaging of Lithium Vacancies in a Battery Cathode by Multislice Electron Ptychography
Source: Nano Lett. 2026 May 29;26(23):7535–42. doi: 10.1021/acs.nanolett.6c00786 (PMC13281515; doi:10.1021/acs.nanolett.6c00786)
Supplement: Supplementary file 1 [file nl6c00786_si_001.pdf]

Supporting Information

Atomic-scale imaging of lithium vacancies in a battery cathode by multislice electron ptychography

Dasol Yoon[1], Harikrishnan KP [2], Eleanor Richard[2], Yu-Tsun Shao[3], Yao Yang[4], Hector D. Abruña[4], David A. Muller[1,2,5,*]

[1]Department of Materials Science and Engineering, Cornell University, Ithaca, NY, 14853, USA

[2]School of Applied and Engineering Physics, Cornell University, Ithaca, NY, 14853, USA

[3]Mork Family Department of Chemical Engineering and Materials Science, University of Southern California, Los Angeles, CA, 90089, USA

[4]Department of Chemistry and Chemical Biology, Cornell University, Ithaca, NY 14853, USA

[5]Kavli Institute at Cornell for Nanoscale Science, Cornell University, Ithaca, NY, 14853, USA

Corresponding author email: david.a.muller@cornell.edu

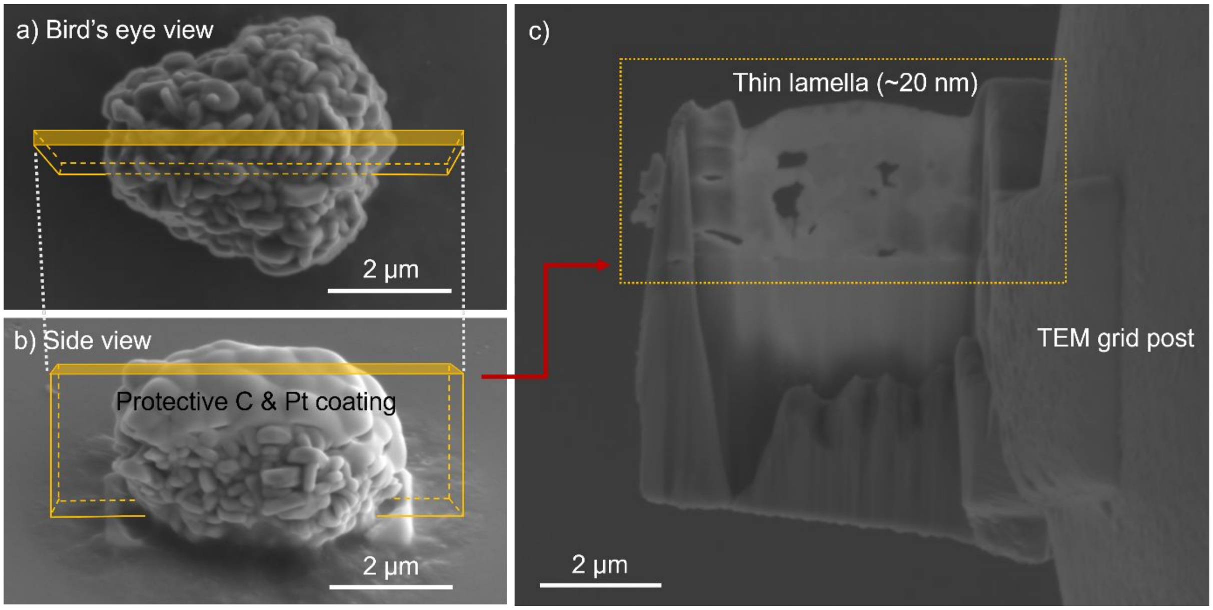

Supplementary Fig. 1. SEM images of the NMC-111 secondary particle for the TEM lamella preparation. (a) Bird's eye view of the particle before putting a protective coating on top. (b) Side view of the particle after putting protective C and Pt coatings on. (c) Thin lamella of the sample attached to the TEM grid post after the FIB milling process.

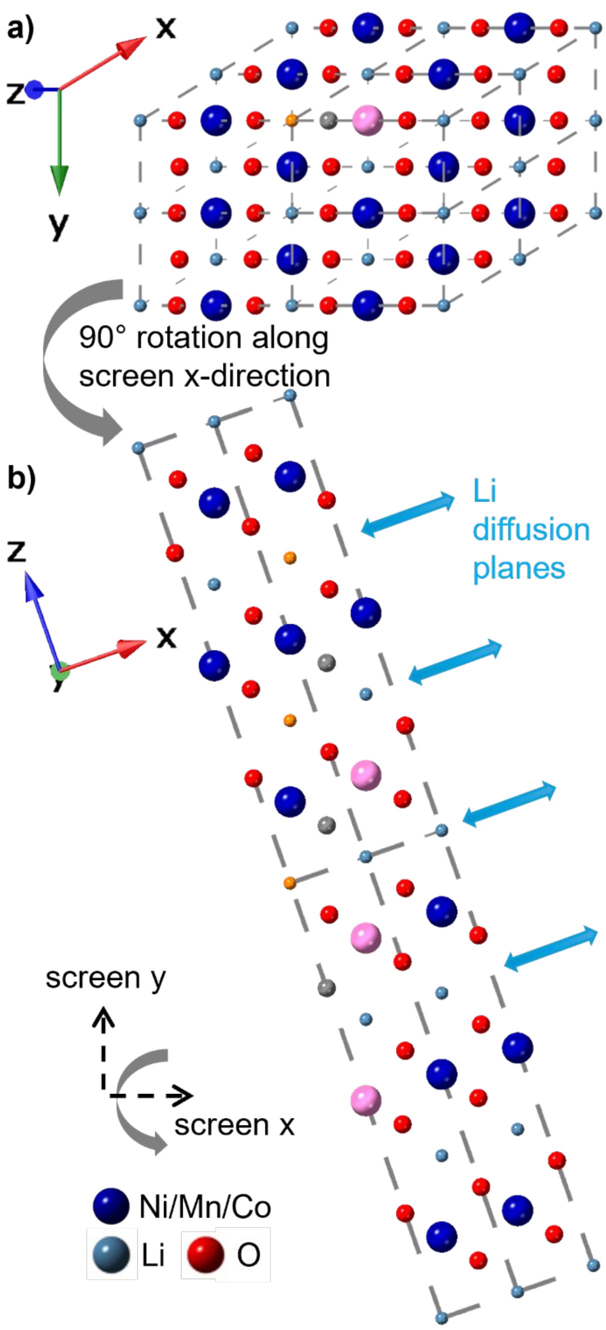

Supplementary Fig. 2. Atomic model of Li-NMC-111 viewed down (a) [211] and (b) [010] zone axes. Some Li (orange), O (gray), and Co (pink) atoms are colored differently as a guide for the eye.

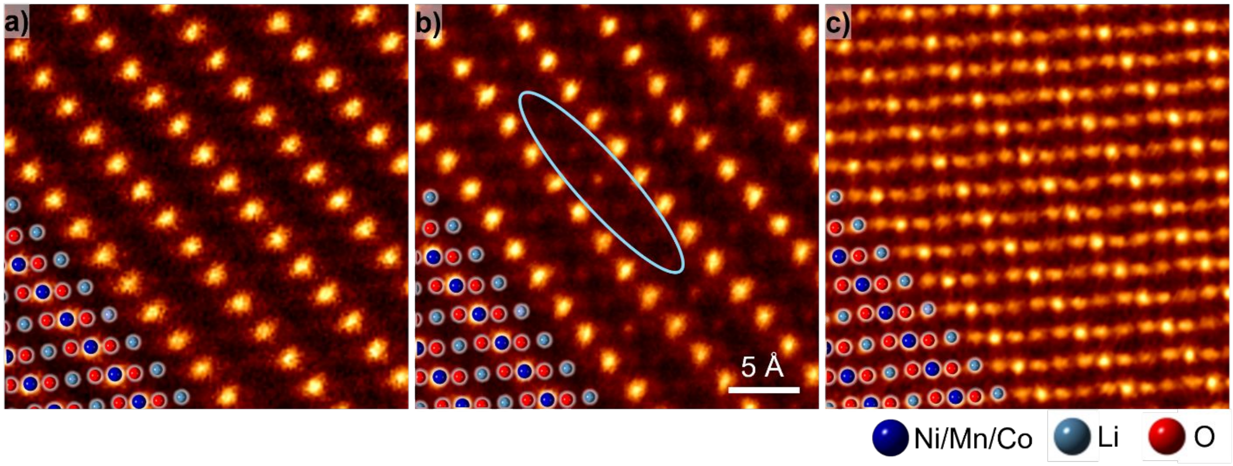

Supplementary Fig. 3. (a, b) HAADF and (c) MEP images of NMC-111 viewed down the [110] axis. Blue oval in (b) denotes some of Li sites occupied by transition metal atoms due to the knock-on damage. (c) The contrast in intensities between heavy transition metal atoms and the light Li and O atoms is not great due to the knock-on damage. Note that experimental conditions could be further optimized to minimize the beam damage along the [110] as well.

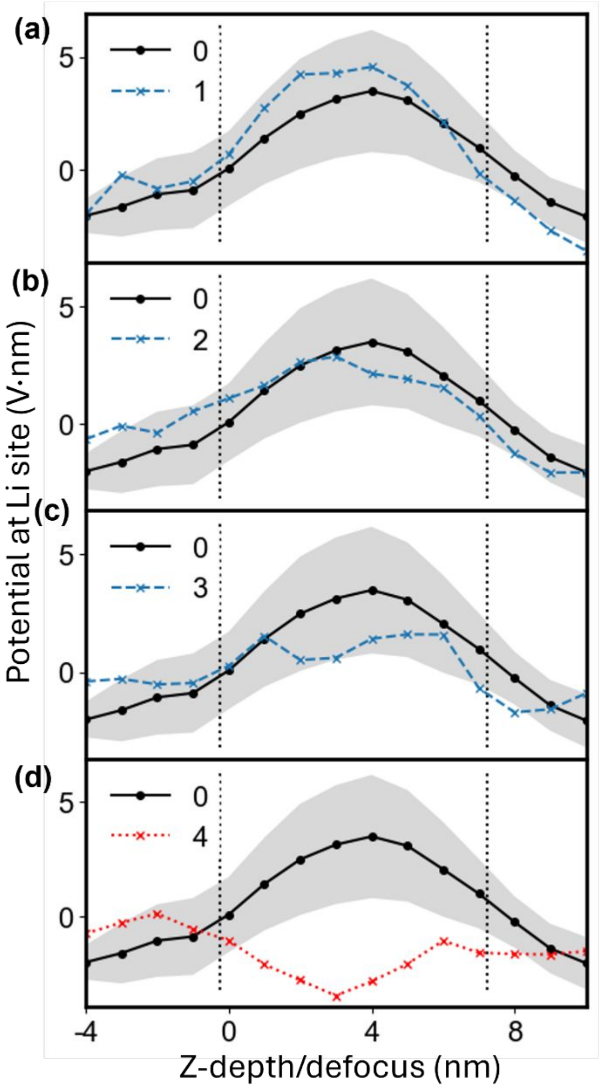

Supplementary Fig. 4. Atomic column profiles of an MEP reconstruction from simulated data along lithium atom columns with no vacancy (black solid line) and vacancies (dashed lines). To account for thermal vibrations, the atom columns were simulated using a frozen-phonon model with a standard deviation of 0.1 Å along x, y, and z. The shaded area indicates the standard deviation of 8 atomic column profiles along fully filled Li atom columns. In panels (a-c), corresponding to one, two, and three consecutive vacancies, the dashed lines remain within the shaded region at the given dose of 5 × 10^4 e·Å^{-2}. The profiles, with up to three vacancies, are hard to tell apart from the fluctuations in fully filled column profiles. In panel (d), the dotted profile corresponding to four vacancies in a row is clearly distinguishable. Note that these values depend on the experimental conditions like dose and convergence angle. Because MEP is linear in the projected potential [13], lateral displacements mainly reduce the peak intensity and broaden the column while conserving the column-integrated intensity, whereas vacancies reduce both the peak and the column-integrated intensity. Random

displacements with standard deviations $\gtrsim 0.13$Å should be detectable as column broadening.

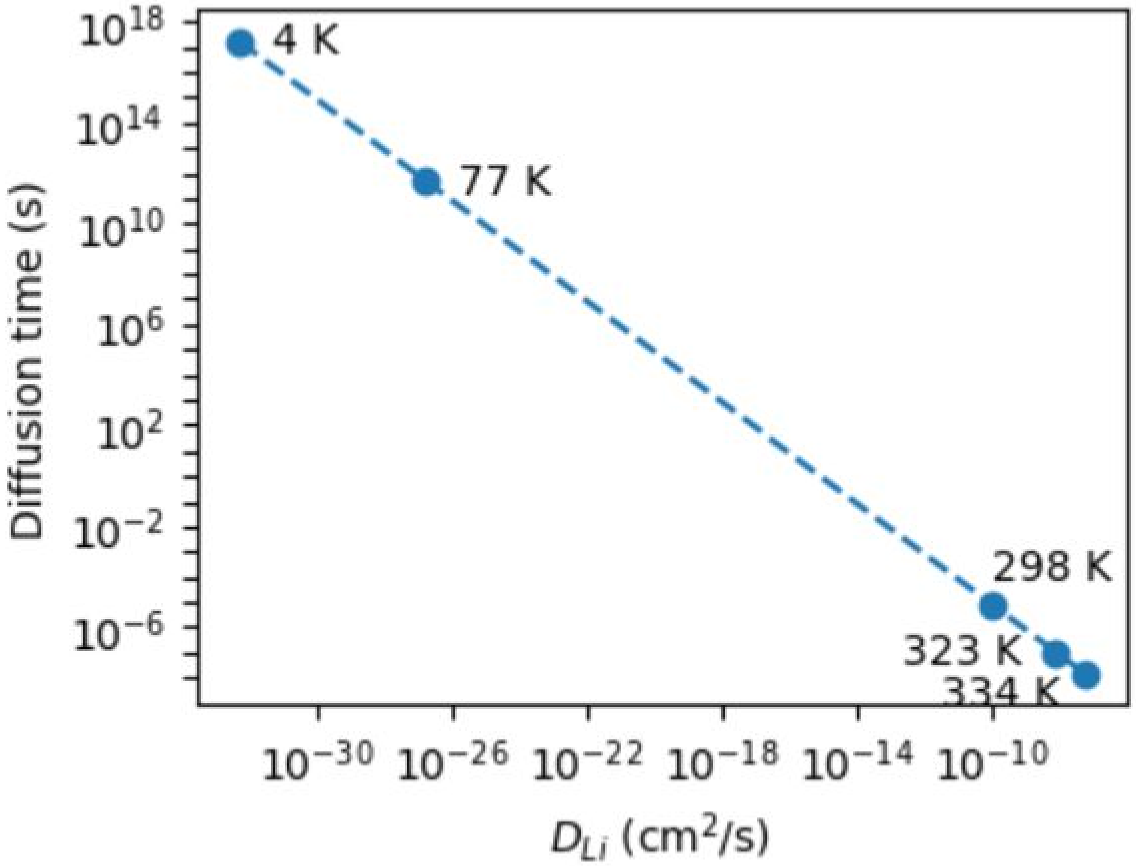

Supplementary Fig. 5. Diffusion coefficients for Li ions at 4K and 77K, extrapolated using the previously reported values at temperatures ranging from 298 K to 334 K. [S1]

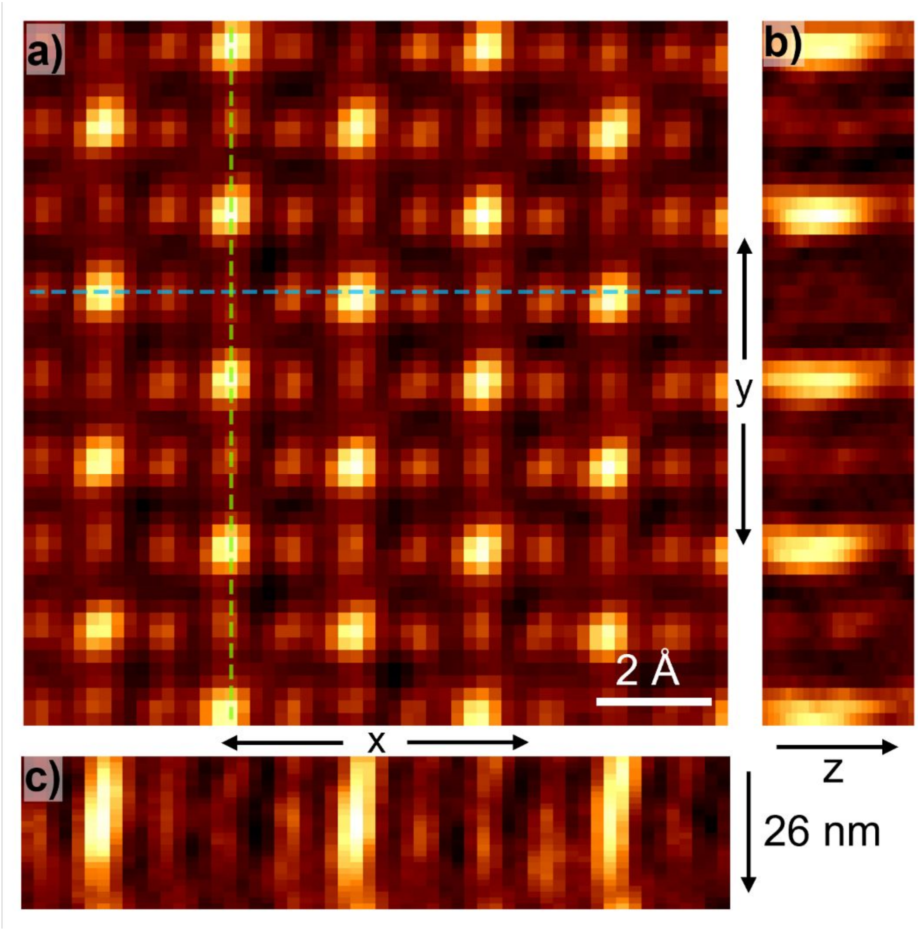

Supplementary Fig. 6. MEP reconstruction on data taken at cryogenic liquid nitrogen temperature. (a) Reconstructed image slices summed up along the depth direction. (b, c) Depth profiles along the lines of atoms denoted with green and blue dotted lines, respectively.

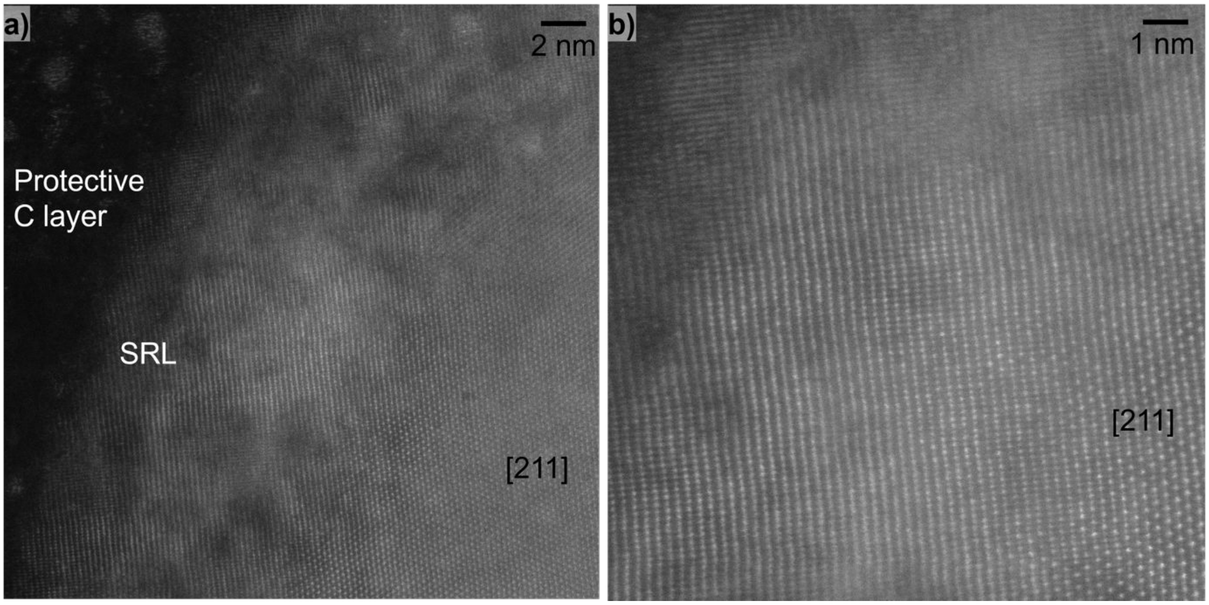

Supplementary Fig. 7. (a, b) HAADF images showing SRL regions on the left and [211] bulk phase of the primary particle on the right. (b) HAADF image with a higher magnification to show the atom columns better.

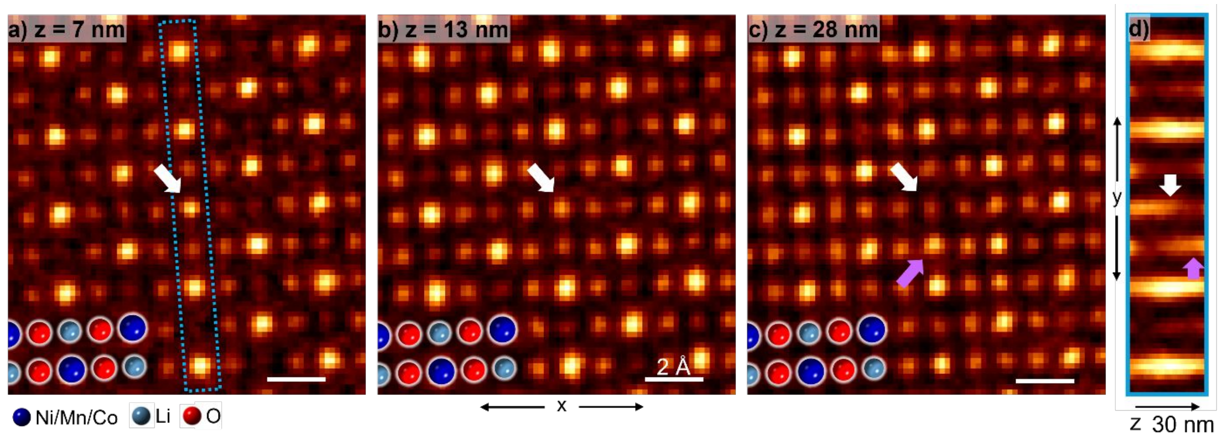

Supplementary Fig. 8. (a-c) Depth slices at 7, 13, and 28 nm into the sample. (d) Depth profile along atom columns enclosed in the blue box in (a). White arrow denotes the NMC column whose atoms get depleted toward the bottom of the sample and replace the nearby Li site pointed with a purple arrow, which is reflected in the decreasing and increasing intensities of the respective atom columns.

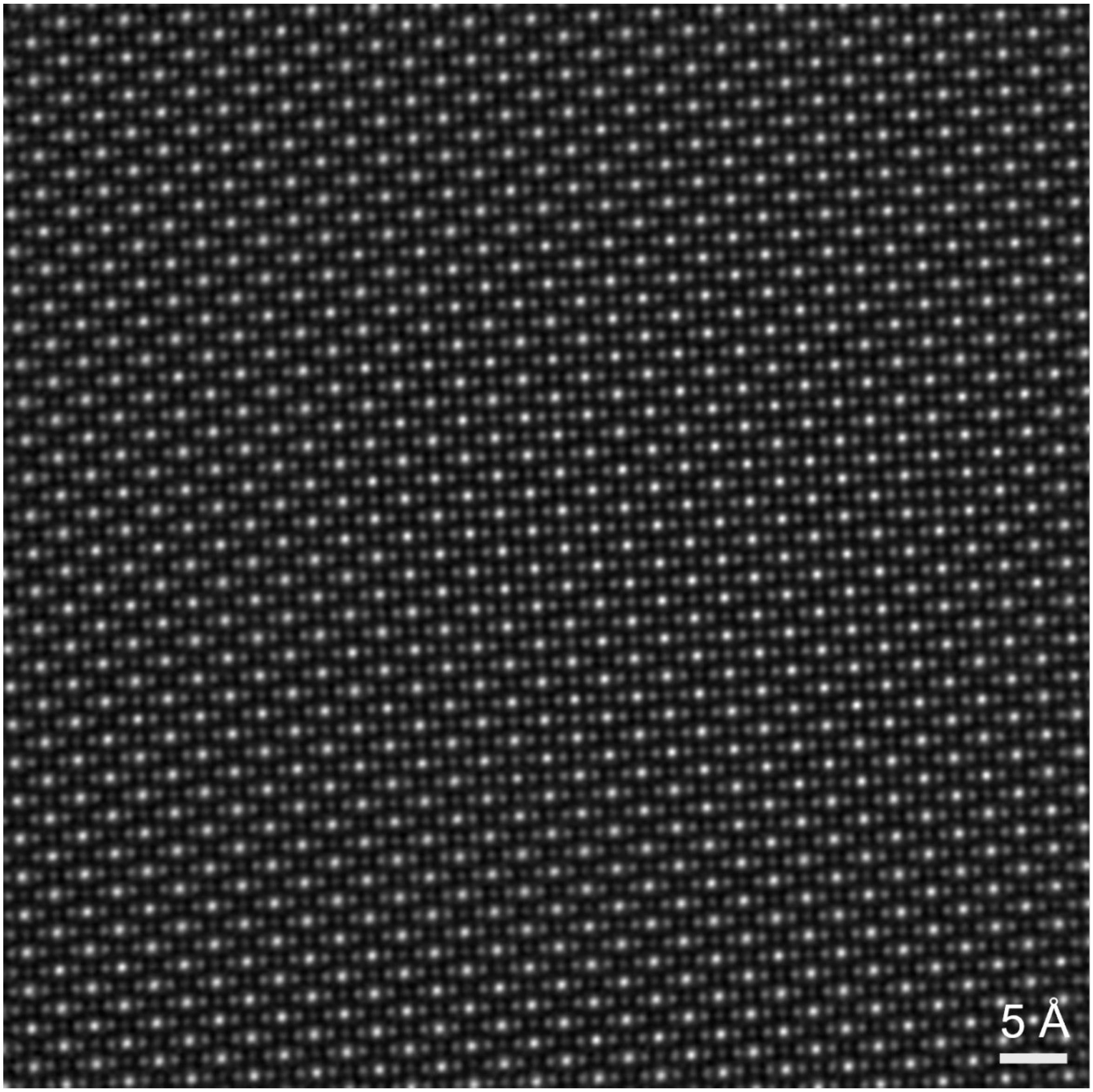

Supplementary Fig. 9. An image of an MEP reconstructed layers summed along the depth direction, showing the full field of view from a single scan dataset. Figure 2 shows a part of this dataset.

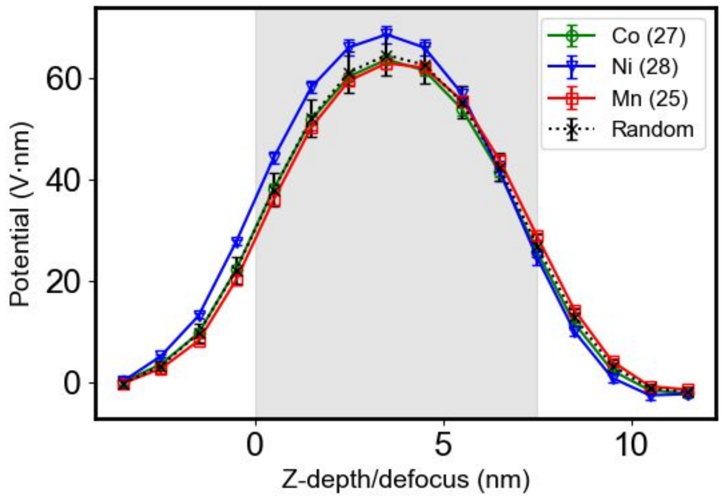

Supplementary Fig. 10. Atomic column profiles from an MEP reconstruction of simulated data for transition metal atom columns containing only Co (green), only Ni (blue), only Mn (red), and an equal mix of Ni/Mn/Co (1:1:1; black). The simulation and reconstruction conditions are the same as in the other simulations, with convergence angle of 21.4 mrad and dose of 5×10^4 e·Å^{-2}.

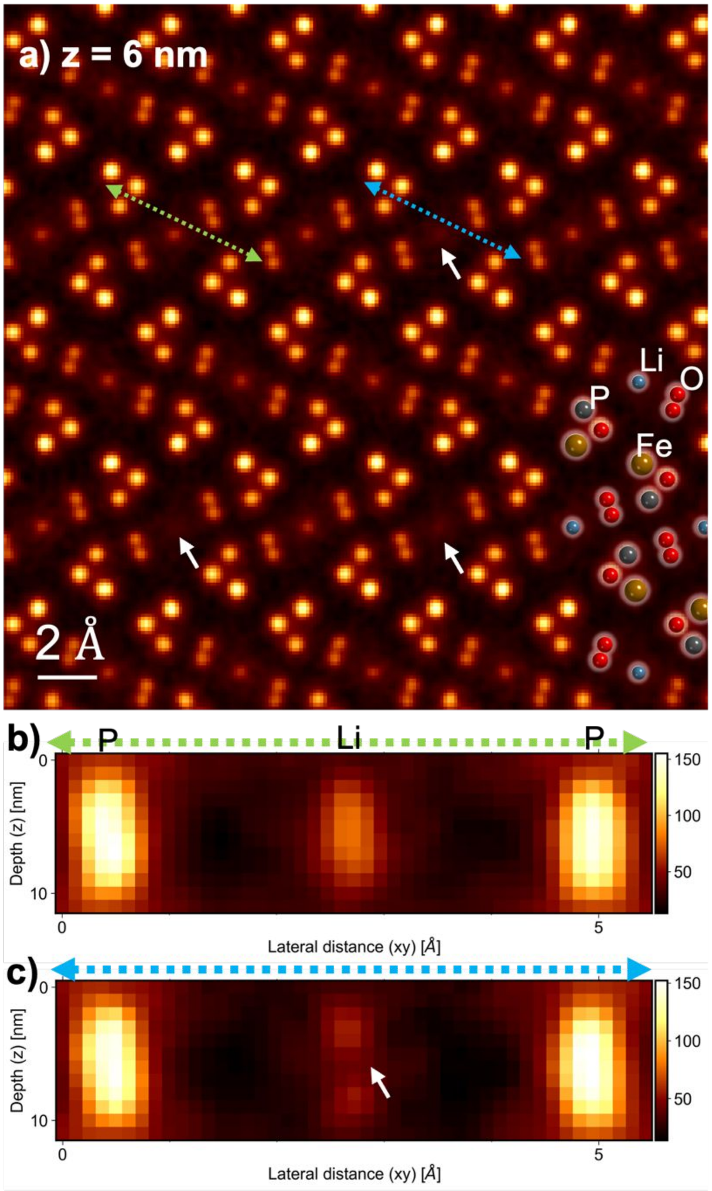

Supplementary Fig. 11. (a) Multislice electron ptychography reconstructed slice at a depth of 6 nm from a simulated $LiFePO_4$ dataset imaged down the [010] zone axis. The probe convergence semi-angle is 30 mrad and the electron dose is 8.4×10^4 e·Å^{-2}. (b,c) Depth profiles along the row of atomic columns indicated with (b) green and (c) blue dashed lines in (a). Li-site vacancies are marked with white arrows.

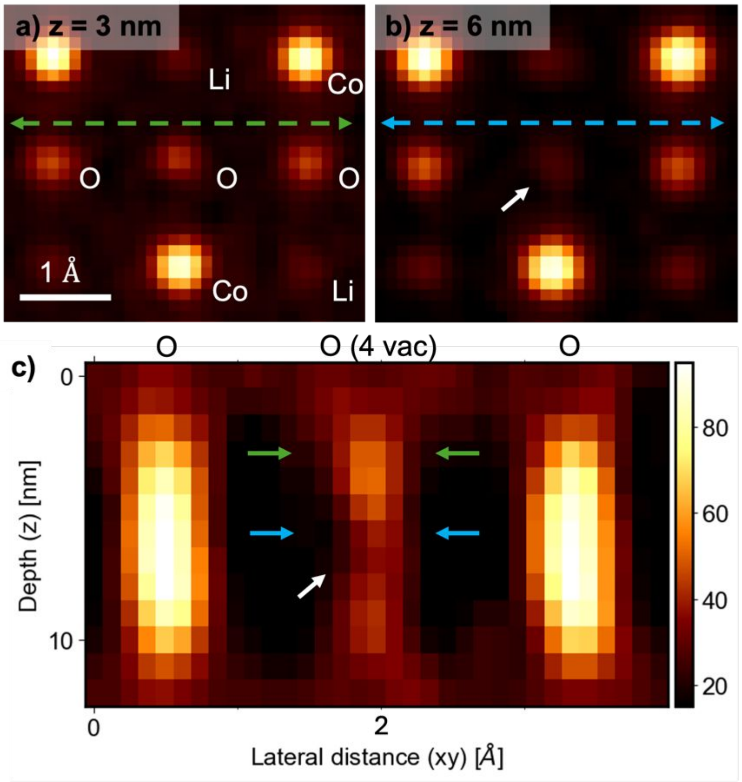

Supplementary Fig. 12. MEP reconstructed slices at depths of (a) 3 nm and (b) 6 nm from a simulated $LiCoO_2$ along [211] zone axis. (c) Depth profile along the row of atomic columns indicated by the dashed lines in (a,b). The central O column contains 4 vacancies (white arrow). Dose used for this simulation is 5×10^4 e·Å^{-2}.

[S1] R. Amin and Y.-M. Chiang, “Characterization of Electronic and Ionic Transport in $Li_{1-x}Ni_{0.33}Mn_{0.33}Co_{0.33}O_2$ (NMC_{333}) and $Li_{1-x}Ni_{0.50}Mn_{0.20}Co_{0.30}O_2$ (NMC_{523}) as a Function of Li Content,” *J. Electrochem. Soc.*, vol. 163, no. 8, pp. A1512–A1517, 2016, doi: 10.1149/2.0131608jes.
